# Overcoming resistance to PD-1/PD-L1 inhibitors in esophageal cancer

**DOI:** 10.3389/fonc.2022.955163

**Published:** 2022-09-05

**Authors:** Chao Cheng, Lingdun Zhuge, Xin Xiao, Siyuan Luan, Yong Yuan

**Affiliations:** ^1^ Department of Thoracic Surgery, West China Hospital of Sichuan University, Chengdu, China; ^2^ Department of Head and Neck Surgery, National Cancer Center/National Clinical Research Center for Cancer/Cancer Hospital, Chinese Academy of Medical Sciences and Peking Union Medical College, Beijing, China

**Keywords:** esophageal cancer, immunotherapy, resistance, immune checkpoint inhibitors, programmed death 1, programmed death-ligand 1

## Abstract

As the predominant treatment option of the immunotherapy for advanced esophageal cancer (EC), the application of programmed death 1 (PD-1) and programmed death-ligand 1 (PD-L1) inhibitors brings new hope to clinical practice. However, a considerable portion of patients do not response to this therapy, meanwhile most patients sensitive to PD-1 or PD-L1 antibody initially will develop resistance to the treatment eventually. To break through the limits of clinical effect, it is of critical importance to make a profound understanding of the mechanisms of so called primary resistance and acquired resistance. Subsequently, exploring potent predictors to identify suitable patients for anti-PD-1/PD-L1 treatment and investigating efficient strategies to overcome drug resistance will be helpful to expend the benefit of immunotherapy. In the present view, we summarized the potential predictive factors for anti-PD-1/PD-L1 immunotherapy in EC, and demonstrated the plausible mechanisms of resistance to PD-1/PD-L1 blockade as well as its feasible solutions.

## Introduction

Esophageal cancer (EC) is the 6^th^ leading cause of cancer related death worldwide (1). The treatment for EC mainly depends on surgery, chemotherapy and radiotherapy, but the prognosis remains unfavorable (2). Recently, with a deeper understanding of cancer related immune mechanisms, immunotherapy has been widely studied and has brought promising therapeutic outcomes (3–5). Programmed death-1 (PD-1) and programmed death-ligand 1 (PD-L1) are regarded as a pair of critical immune checkpoints, by which cancer cells can suppress the activity of effective immune cells, allowing the immune escape of cancer (6). PD-1/PD-L1 blockade, one of the most efficient immune checkpoint inhibitors (ICIs), is designed to inhibit the interaction between PD-1 and PD-L1, helping to restore the anti-cancer immune response, which was approved by USFDA as a first-line treatment for advanced EC. Despite the compelling outcomes of anti-PD-1/PD-L1 therapy, drug resistance is regarded as a major problem of this treatment, since a majority of patients do not have a response to ICIs at the beginning of the therapy (7, 8), and those who are sensitive to ICIs will eventually develop therapeutic resistance (9, 10). Thus, a profound understanding of resistance to PD−1/PD−L1 blockade is of necessity to enhance the therapeutic effect of ICIs for patients with EC. In this review, we summarized the main mechanisms of resistance to anti-PD−1/PD−L1 treatment and provided some reliable predictors for the treatment, hoping to find out directions to overcome drug resistance.

## Mechanisms of resistance to PD−1/PD−L1 blockade

**Figure 1:**
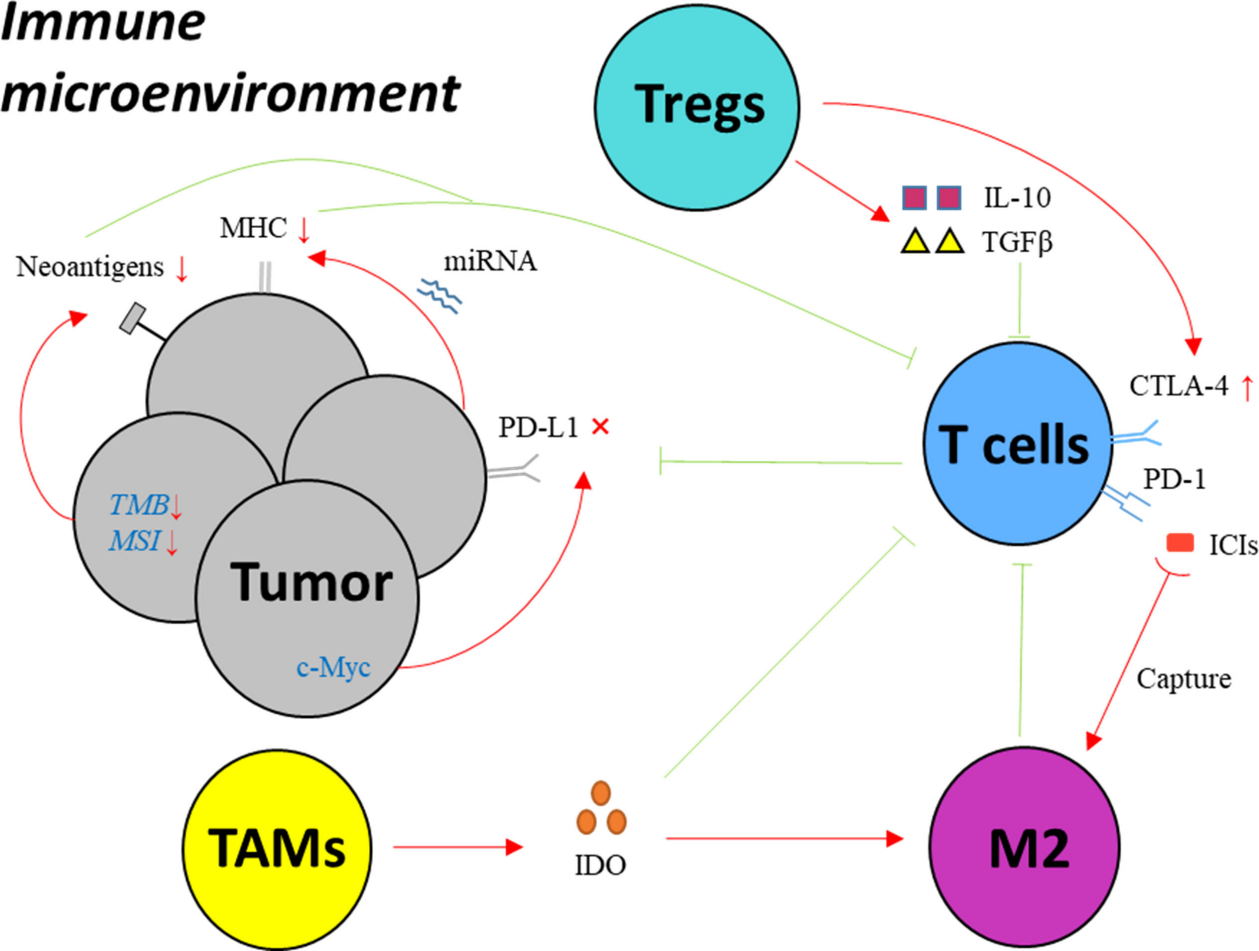
Key mechanisms of drug resistance to PD-1/PD-L1 inhibitors in EC. MHC: major histocompatibility complex; PD-1, programmed cell death protein 1; PD-L1, programmed death-ligand 1; IFN-γ, interferon gamma; CTLA-4, cytotoxic T-lymphocyte-associated protein 4; Treg, regulatory T cell; IDO, indoleamine 2,3-dioxygenase; TAM, tumor associated macrophage; M2, macrophages with M2 phenotype; ICI, immune checkpoint inhibitor; miRNA, microRNA; TMB, tumor mutation burden; MSI, microsatellite instability; TGF-β, transforming growth factor-β; IL-10, interleukin-10.

The interaction of PD-1 with its corresponding ligand PD-L1 leads to the disability of effective T cells, by which cancer cells manage to evade the surveillance and attack from immune system (6). PD−1/PD−L1 inhibitor immunotherapy aims to block PD-1 or PD-L1 expressed on cell surface in order to activate T cells. However, a majority of cancer patients have no significant response to PD−1/PD−L1 targeted treatment (7–10), since cancer cells develop diverse mechanisms to resist ICIs. (**Figure 1** and **Table 1**)

**Table 1:** The common mechanisms of resistance to PD-1/PD-L1 inhibitors in EC and other cancers.

	Common causes	EC specific
Aberrant PD-L1expression	JAK1/2-inactivating mutations Down-regulation of PD-L1 expression Methylation of PD-L1 promoter	Alteration of c-Myc expression PD-L1 expression altered by various microenvironment
Aberrant neoantigen expression	low TMB, low MSI, MMR	
Aberrant neoantigen presentation	Decreased expression of β2M Loss of functional β2M	MHC regulated by miRNAs
Suppressive microenvironment	Immunosuppressive chemokines and cytokines Immune cells: Tregs, TAMs, etc	

### Aberrant expression of PD-L1

Blocking the interaction between PD-1 and PD- L1 is the aim of anti-PD-1/PD-L1 immunotherapy, therefore its therapeutic effect depends on the expression of PD-1 or PD-L1 in cancer microenvironment (11, 12). The abundance of PD-L1 was reported to be related to the genetic signature of cancer cells. For instance, in the experiments based on melanoma cell lines, JAK1/2-inactivating mutations resulting in the lack of reactive PD-L1 expression, lead to the primary resistance to PD−1/PD−L1 inhibitors (13). In addition, in lung cancer, drug resistance of cancer cells was found to be induced after anti-PD-1 therapy by down-regulation of PD-L1 expression and methylation of PD-L1 promoter (14). A series of studies have revealed plausible explanations for the mechanisms of PD-L1 regulation in EC, such as the alteration of PD-L1 level by c-Myc expression (15), and the changeable PD-L1 expression caused by various immune microenvironment (16), helping to explore solutions to overcome the resistance to PD−1/PD−L1 inhibitors for EC patients.

### Attenuated expression and presentation of tumor neoantigens

Neoantigens produced by cancer cells are indispensable factors for the proliferation and activation of T lymphocytes. The absence of neoantigens disables the recognition of cancer cells by CD8^+^ T cells, leading to impaired anti-cancer immunity (17). A straightforward way to elude the recognition from immune cells is that cancer cells evolve to lose its neoantigens on the surface. The expression of neoantigens is considered to be related with tumor mutation burden (TMB), since the accumulation of gene mutation creates tumor productions differentiated enough from normal tissue, triggering the response of T cells (18). Base mismatches during the DNA replication process are routinely fixed by some gene components, known as mismatch repair (MMR) genes. But in cancer cells, the deficient MMR results in the occurrence of microsatellite instability (MSI), allowing the accumulation of gene mutations (19, 20). In another word, low TMB level, MSI-low and MMR represent diminished immunogenicity and poor effect of anti-cancer immunotherapy, and are considered to promote primary resistance.

The presentation of tumor neoantigens, another key factor for immunological recognition, mainly relies on the major histocompatibility complex (MHC). Cancer develops an immune escape strategy by down-regulation of MHC class I expression which induces the dysfunction of CD8^+^ T cells. Beta-2-microglobulin (β2M) as an essential component of MHC class I molecule, helps to present tumor antigens on cell membrane. Cancer cells interfere the synthesis of MHC class I molecule through decreased expression of β2M and loss of functional β2M as a response to immunotherapy, contributing to the acquired resistance (21, 22), which was illustrated by several studies of melanoma. New evidence of MHC-I regulation process has been uncovered by recent studies. For instance, it was reported that reduced expression of MHC-I in esophageal adenocarcinoma (EAC) can be caused by the increased levels of MIR125a-5p and MIR148a-3p, which influenced therapeutic effect (23).

### Immune suppressive microenvironment

Immune microenvironment is significantly correlated with prognosis of cancer patient. The interaction among cancer cells, immune cells and immune molecules presents distinctively different immune phenotypes of cancer, having a conspicuous influence on the outcomes of anti-cancer treatment including anti-PD-1/PD-L1 immunotherapy.

Regulatory T cells (Tregs), a typical type of immunosuppressive cells, play a role in immune tolerance maintenance and preventing anti-cancer immune responses through suppressing activation of T cells and APCs, which consequently reduces the effect of ICIs (24). The relevant mechanisms are complex, such as up-regulation of CTLA-4 and increasing expression of PD-L1 on cell surface (25). Additionally, a variety of suppressive cytokines produced by Tregs like IL-10 and TGFβ, act on T lymphocytes as well as other immune cells and then hinder their activation (26).

The function of tumor-associated macrophages (TAMs) polarizes into either anti-tumoral or pro-tumoral effect, known as M1 and M2 subtype (27). Latest studies have demonstrated the roles of TAMs in tumor resistance to PD−1/PD−L1 blockade as follow. TAMs secret a certain type of molecules with immunosuppressive effect, named indoleamine 2,3-dioxygenase (IDO), deactivating effective T cells and inducing polarization of TAMs towards M2 subtype (28). More interestingly, TAMs were found to sabotage the combination of ICI with its target by capturing PD−1 antibody from T cell surface, preventing reactivation of dysfunctional T cells (29).

Immunosuppressive chemokines and cytokines also promote the resistance to anti-PD-1/PD-L1 immunotherapy. TGF-β, a pivotal molecule maintaining immune tolerance, has been reported to shape the microenvironment and restrain the effect of PD-L1 blockade by restricting T-cell infiltration (26). Others like CCL2, CCL22, CCL5, CCL7 and CXCL8, also take part in limiting the efficacy of ICIs.

## Predictors for PD-1/PD-L1 inhibitor effect

**Figure 2:**
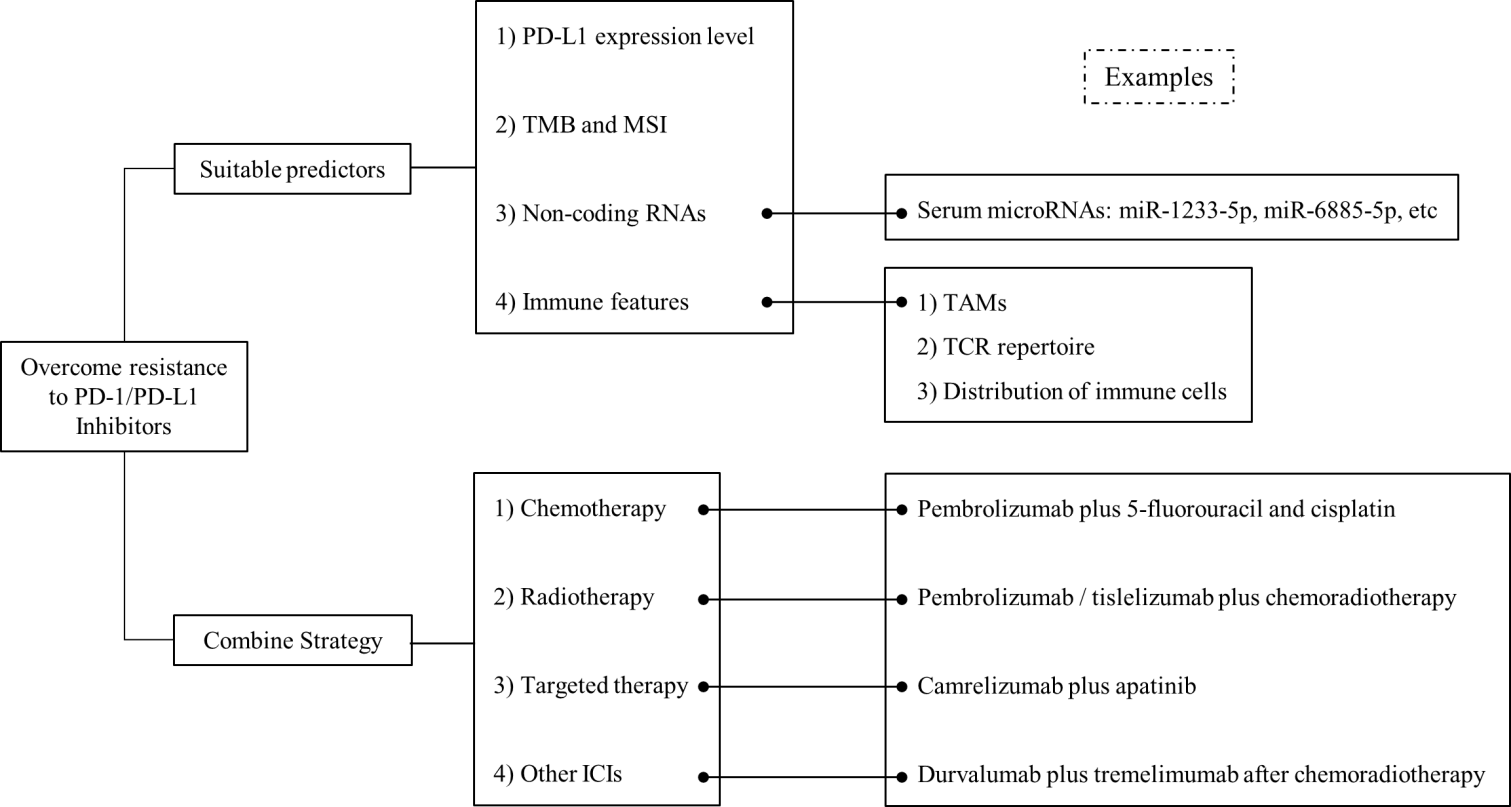
Overview on the strategies to overcome drug resistance to PD-1/PD-L1 inhibitors in EC. Strategies including suitable predictors and combined therapy, have been proposed to improve the effect of PD-1/PD-L1 inhibitors in EC.

The response to ICI treatment varies according to different condition of each individual. Therefore, it is critical to identify reliable indicators for accurate prediction of ICI efficacy and precise identification of suitable patients for immunotherapy. At present, widely used predictors include PD-L1 expression level, TMB, MSI and so forth. With the further understanding of immune mechanisms, more effective biomarkers can be identified for ICI treatment (**Figure 2**).

### PD-L1 expression level

PD-L1 expression level in tumor tissue is widely used in clinical practice as an indicator for the therapeutic effect of anti-PD-1/PD-L1 treatment. Patients with high expression of PD-L1 are supposed to have better prognosis after receiving anti-PD-1/PD-L1 treatment (11, 12). In EAC, according to outcomes of several well-known clinical trials, the lack of benefit of ICIs was observed in the low PD-L1-expressing subgroup (30). However, its predictive efficacy is not that satisfactory, since some patients with low expression of PD-L1 have a positive response to ICIs (31), and vice versa. Additionally, the expression level of PD-L1 does not remain constant. It can be altered during the therapeutic course. A recent study demonstrated the profound influence on the expression of PD-L1 and PD-L2 by chemotherapeutic agents in esophageal squamous cell carcinoma (ESCC) (32), which implied one-time evaluation of PD-L1 might not be sufficient to predict the efficacy of ICIs.

### TMB and MSI

The predictive value of TMB and MSI for anti-PD-1/PD-L1 treatment has been verified (33–35). In many malignancies, including EC, high level of TMB or MSI is positively correlated with the prognosis of patients receiving ICIs. The predictive role of TMB is found to be independent of PD-L1 expression level by recent studies (36). Therefore, the approved indications of the application of ICIs include TMB-high or MSI-high EC.

### Non-coding RNAs

It is widely acknowledged that non-coding RNAs, such as microRNAs, long non-coding RNAs, and circular RNAs, are involved in multiple cellular functions. Their predictive value for effect of ICIs is gradually revealed by a growing number of studies.

New evidence showed that a group of MDSC-relevant microRNAs indicating MDSC activity closely relates with resistance to treatment with ICIs. A recent study suggested that these RNAs might be potential blood biomarkers predicting immunotherapy outcomes in melanoma (37). A recent study based on a phase II clinical trial investigated the value of microRNAs as predictive makers, and found that serum microRNAs, including miR-1233-5p, miR-6885-5p, miR-4698 and miR-128-2-5p, capable of predicting the response to nivolumab in patients with advanced EC (38).

### Immune features

The immune characteristics of cancer patients are significantly related with the outcomes of ICI treatment. T cell receptor (TCR) repertoire has been widely studied as a predictor for immunotherapy efficiency. In EC, researchers found the peripheral CD8^+^ TCR diversity at baseline and dynamic alteration of intratumoral TCRs showed significant correlation with the prognosis of radiotherapy combined with immunotherapy (39). More interestingly, the spatial distribution patterns of immune cells were recently identified as prognostic factors for the therapeutic effect of ICIs in EC, since the spatial distance between cancer cells and various subtypes of immune cells, such as dendritic cells and macrophages, is correlated with PFS and OS (40). The infiltration of macrophages in cancer microenvironment is recognized as an indicator of poor prognosis of EC. Previous studies have identified the role of TAMs in increasing PD-L1 expression in EC (41). Given the close correlation between TAMs and PD-L1, a clinical trial was launched to investigate the therapeutic efficacy of combination of CSF‐1R blockade (TAM-targeting therapy) with PD‐1/PD‐L1 inhibitor in several advanced solid tumors, such as lung cancer, and pancreatic cancer (42).

## Strategies to overcome drug resistance and future directions

To enhance the efficacy of ICIs, combination strategy is adopted (**Figure 2**). Immunotherapy combined with chemotherapy is the most widely used method to improve therapeutic effect for EC patients, since the outcomes of KEYNOTE-590 showed significantly improved survival in ESCC patients when pembrolizumab was added to chemotherapy (43). The plausible explanations for the improved outcomes of the combination might involve several mechanisms, such as increased sensitivity of cancer cells to immunotherapy *via* increase of mutation burden, upregulation of PD-L1 expression, and restoration of exhausted immune cells by chemotherapeutic agents (44, 45). Recently, the value of radiotherapy in addition to ICIs has attracted some attention. Previous study has revealed the immune-related effects of radiotherapy in cancer treatment, including EC (46, 47). For instance, the death of cancer cells allows more exposure of tumor specific antigens, activating antigen-presenting cells (48). A series studies such as KEYNOTE-975 and RATIONALE 311, have been designed to explore the therapeutic effect of ICIs plus chemoradiotherapy in EC treatment (49, 50). Anti-PD-1/PD-L1 treatment added to targeted therapy is regarded as a promising direction. Clinical trials have been launched to evaluate the integration of ICIs and targeted therapy. In patients with advanced ESCC, a single-arm, phase II study analyzing the safety and the efficacy of camrelizumab plus apatinib as second-line treatment showed 34.6% of patients had an objective response, and 44% of patients had grade 3 or worse adverse events (51). The promising activity and manageable toxicity of the combination treatment indicated it might be an option for patients with advanced ESCC. The effective combination of PD-1/PD-L1 blockade with other type of ICIs is under exploration in several malignancies including EC. A latest research reported that additional application of durvalumab and tremelimumab after chemoradiotherapy significantly improved survival in patients with locally advanced ESCC, especially in those with PD-L1 positive tumors (52). Additionally, some studies have found the combination of anti-PD-1/PD-L1 inhibitors with some chemokine or cytokine blockades might bring new solutions to overcome drug resistance. For instance, a novel type of antibody, named YM101, was developed to enhance the effect of ICIs by blocking PD-1/PD-L1 and TGF-β simultaneously, which was found to have a superior anti-tumor effect compared to the monotherapies (53). Some new emerging combination strategies exhibited potent antitumor efficacy (54), which might be future direction for cancer immunotherapy. For example, combining Mn^2+^ with YM101 has a synergistic antitumor effect, effectively controlling tumor growth and prolonging the survival of tumor-bearing mice (55). This novel cocktail strategy has the potential to be a universal regimen for inflamed and non-inflamed tumors.

## Discussion

Drug resistance hinders the applicability of PD-1/PD-L1 inhibitors in the treatment of EC. Although the mechanisms are complicated and multifactorial, a systematic investigation and understanding will undoubtedly contribute to establish new strategies to improve efficacy and outcomes of anti-PD-1/PD-L1 treatment in patients with EC.

Given the evidence that we have gathered, one rational option to avoid resistance against PD-L/PD-1 blockade is to identify the suitable population before the application of the therapy. A thorough and accurate profile of immunological status seems to be necessary for each patient, which is also supposed to be a non-invasive or minimal invasive procedure. The modern technologies, such as new generation sequencing and flow cytometry, facilitate the analysis of immunological profile, providing the feasibility to achieve this goal. Some recent studies illustrated the method to depict the status of immune activities using peripheral blood by flow cemetery. For example, in lung cancer, researchers found this method was a reliable and efficient way to identify candidates who might have a better chance of responding to PD-1/PD-L1 inhibitors (56). Another key point of evaluating the appropriateness of anti-PD-1/PD-L1 treatment is a dynamic monitor of immunological profile for patients, since the fluctuations or changes of immune status can significantly influence the effect of immunotherapy, as well as can help to distinguish the response groups of patients. For instance, some researchers compared the changes of different immune variables in blood samples derived from cancer patients before and after anti-PD-1 treatment, and confirmed these alterations as useful markers to identify eligible patients for anti-PD-1 therapy (57). Of course, the definition of immunological profile is not confined to immune cells or molecules. As aforementioned, the genetic characteristics of cancer cells are also correlated with the anti-cancer immune activities, and therefore have an influence on the outcomes of immunotherapy. With the widely use of new generation sequencing in clinical oncology, it is reasonable to assume this will be a promising approach to screening appropriate candidates for ICI treatment. However, our current knowledge of the correlation between genetic features and outcomes of anti- PD-1/PD-L1 therapies is limited. Hence, identification and construction of gene panels affecting the pathways of immune checkpoints and the outcomes of ICI treatment will play an indispensable role as a critical research subject in oncology in the near future.

In cancer microenvironment, there is an equilibrium between conditions that promote and suppress anti-cancer immunity, which is described as a conceptual framework named “cancer-immune set point” (58). It works as a presumption helping to interpret changeable response to ICI therapies. From this perspective, the purpose of current combination therapies adopted to overcome drug resistance against PD-1/PD-L1 inhibitors can be considered as strategies to enhance the factors contributing to anti-cancer immunity by increasing the expression of neoantigens and further activation of T cells, as well as to diminish unfavorable factors by regulating suppressive immune cells and chemokines. Take the combination of chemotherapy and anti-PD-1/PD-L1 inhibitors as an example. Chemotherapy can cause the up-regulation of antigen expression by triggering DNA damage of cancer cells on one hand, on the other hand it was reported that chemotherapeutic agents are able to alter immune microenvironment of EC through various methods, such as upregulation of cell surface PD-L1 expression (59). And this type of combination showed promising clinical outcomes. Other proposal of combination strategies can be inspired and designed following this thread of thought. Some studies have demonstrated the role of HER2 antibody in promoting anti-cancer immune activities (60), leading to the logical attempt to add anti-HER2 treatment to ICI in EC, which brings us the well-known clinical trials such as KEYNOTE-811 and MAHOGANY studies. With new findings of immune pathways and mechanisms, there is no doubt that more and more efficacious combination strategies will be developed to improve the therapeutic effect of PD-1/PD-L1 inhibitors in EC.

## Conclusions

A clear understanding of the mechanisms of drug resistance and identification of reliable predictors help to develop feasible strategies to overcome resistance to anti-PD-1/PD-L1 treatment, and to improve the therapeutic effects of ICIs in EC.

## Author contributions

CC and LZ have contributed equally. CC, conception, manuscript preparation, data collection, manuscript editing and manuscript review. LZ, conception, manuscript editing and manuscript review. XX, manuscript editing and manuscript review. SL, manuscript review. YY, conception, manuscript editing and manuscript review. All authors contributed to the article and approved the submitted version.

## Funding

This study was supported by the National Natural Science Foundation of China (81802273,81970481), 1.3.5 project for disciplines of excellence, West China Hospital, Sichuan University (Grant No. 2020HXFH047 and 20HXJS005) and Sichuan Science and Technology Program (2022YFS0048).

## Conflict of interest

The authors declare that the research was conducted in the absence of any commercial or financial relationships that could be construed as a potential conflict of interest.

## Publisher’s note

All claims expressed in this article are solely those of the authors and do not necessarily represent those of their affiliated organizations, or those of the publisher, the editors and the reviewers. Any product that may be evaluated in this article, or claim that may be made by its manufacturer, is not guaranteed or endorsed by the publisher.
